# Complete response to talazoparib in patient with pancreatic adenocarcinoma harboring somatic PALB2 mutation: A case report and literature review

**DOI:** 10.3389/fonc.2022.953908

**Published:** 2022-09-02

**Authors:** Andrei Kachmazov, Larisa Bolotina, Anna Kornietskaya, Olesya Kuznetsova, Maxim Ivanov, Alexander Fedenko

**Affiliations:** ^1^ P. Hertsen Moscow Oncology Clinical Research Institute – Branch of the National Medical Research Radiological Centre of the Ministry of Health of the Russian Federation, Moscow, Russia; ^2^ Federal State Budgetary Institution “N.N. Blokhin National Medical Research Center of Oncology” of the Ministry of Health of the Russian Federation, Moscow, Russia; ^3^ RnD Department, Atlas Oncodiagnostics, LLC, Moscow, Russia

**Keywords:** pancreatic cancer, PDAC, talazoparib, PARP inhibitors, PARPi, PALB2, molecular profiling, precision oncology

## Abstract

PARP inhibitors have recently emerged as a maintenance treatment option for metastatic pancreatic cancer patients with germline BRCA mutations. However, the possibility of PARP-inhibitor use as a standalone-targeted therapy for patients with various homologous repair pathway alterations remains mostly undetermined. Here we report a clinical case of a 56-year-old woman with pancreatic ductal adenocarcinoma harboring a somatic PALB2 mutation. Following disease progression after 10 cycles of FOLFIRINOX chemotherapy and two cycles of second-line gemcitabine, she was switched to talazoparib and achieved a complete clinical response after 25 months of treatment. The patient remains alive without clinical or radiological signs of disease progression three years after the start of talazoparib with no targeted therapy-related toxicities. This case highlights the role of broad molecular profiling as a window of opportunity to achieve a durable response for selected pancreatic cancer patients while pinpointing our gaps in understanding the whole picture of management of these patients since a new puzzle element represented by PARP inhibitors was introduced to clinical practice.

## Introduction

Pancreatic ductal adenocarcinoma (PDAC) has historically represented a difficult challenge in oncology practice. It is associated with a poor prognosis, mostly due to most patients presenting with unresectable locally advanced or metastatic disease. While ranking 12th in cancer incidence, PDAC is one of the leading causes of cancer-related mortality worldwide (1). Despite some recent improvements in the early detection and management of this disease, 5-year overall survival still does not exceed 9% (1). Chemotherapy regimens continue to dominate the treatment paradigm for PDAC patients despite the distinctive chemo-resistance of this disease (2). After a long-time struggle to find targets of interest and novel drugs beyond chemotherapy the poly(adenosine diphosphate-ribose) polymerase (PARP) inhibitors came up on the forefront as a possible treatment option for PDAC patients.

PARP inhibitors play an important role in ovarian, prostate, breast, and pancreatic cancer treatment. Though indications vary across cancer types, there are two interconnected major factors guiding the selection of patients for PARP inhibitors: i) evidence of homologous recombination (HR) deficiency-positive status and ii) platinum-sensitive disease. HR deficiency (HRD) impairs the proper maintenance of genomic stability and renders cancer cells vulnerable to inhibited DNA repair proteins, including PARP, as well as DNA-damaging platinum agents.

PDAC is often characterized by germline mutations in genes involved in DNA repair. Approximately 5%–9% of PDAC develop in patients with germline BRCA1/2 and PALB2 mutations. Somatic mutations in HR genes are less common. Somatic BRCA1/2 mutations occur in up to 2% of PDACs, and up to 4% of patients with PDAC harbor a somatic mutation in any HR gene (3). Based on the improvement in progression-free survival that was shown in the randomized phase III POLO trial, olaparib was approved for treating pancreatic cancer patients with germline BRCA mutations whose disease has not progressed after at least 16 weeks of first-line platinum-based chemotherapy. However, the final results of this study revealed no differences in overall survival between olaparib and placebo maintenance groups (4). Further small single-arm Phase II trials demonstrated the clinical activity of rucaparib for the treatment of germline BRCA1 mutated (gBRA1m), gBRCA2m, and gPALB2m PDAC patients without evidence of platinum resistance after 16 weeks of platinum-based chemotherapy. Across 36 patients with measurable disease, ORR was 41.7%, while in the gPALB2m subgroup, three responses were seen across six patients (OR 50%) (5). Talazoparib is another PARP inhibitor approved for treating gBRCAm HER2-negative advanced breast cancer. In *in vitro* studies, talazoparib demonstrated 100-fold higher PARP trapping potency and single-agent cytotoxicity than that of olaparib and rucaparib (6). In phase I, talazoparib showed antitumor activity in gBRCA1m/gBRCA2m PDAC patients with a clinical benefit rate (≥16 weeks) of 31% (four of 13 patients) (7).

Here we present the case of a PDAC patient with a somatic PALB2 mutation who exhibited a dramatic response to talazoparib, illustrating the clinical potential of PARP inhibitors in PDAC outside of approved indications.

## Case presentation

**Figure 1:**
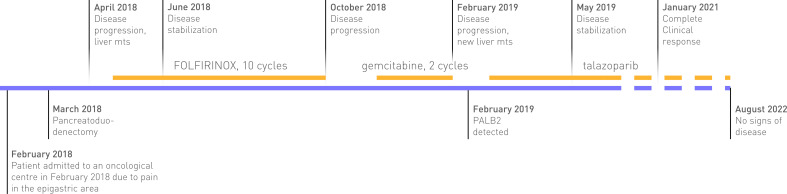
Timeline from first visit to last follow-up.

A 56-year-old woman with no family or personal history of breast, ovarian, prostate, pancreatic, or gastric cancer was admitted to an oncological center in February 2018 because of pain in the epigastric area (**Figure 1**). An initial computed tomography of the abdomen revealed a mass in the head of the pancreas up to 50 mm in the greatest dimension. A biopsy was undertaken and PDAC, grade 2, was confirmed by histological examination. The patient underwent pancreatoduodenectomy with lymph node dissection and cholecystectomy in March 2018. One month later, a PET-CT scan revealed multiple metastatic lesions (up to 16 mm, SUV max 9.3) in both lobes of the liver. First-line systemic therapy was initiated in May 2018. The patient received 10 cycles of the FOLFIRINOX regimen for 6 months, with disease stabilization achieved after the third cycle. Several dose reductions were made throughout treatment because of chemotherapy-induced peripheral neuropathy (CIPN) and febrile neutropenia occurrences despite the use of G-CSF prophylaxis. In October 2018, PET-CT showed disease progression, the appearance of new lesions in the liver, and suspected peritoneal carcinomatosis. Second-line chemotherapy was initiated in December 2018. The patient received two cycles of gemcitabine monotherapy. The first control CT scan in February 2019 showed progression with new metastases in the liver of up to 12 mm.

The primary tumor from the pancreatoduodenectomy was analyzed using a 409-gene panel next-generation sequencing (NGS). The analysis identified a splicing mutation in the PALB2 gene (NM_024675.4: c.3350+1G>T), a nonsense mutation in the BLM gene (NM_000057.4: p.Gln548Ter), and a KRAS G12D mutation. Sanger sequencing confirmed the presence of BLM but not PALB2 variants in peripheral blood lymphocytes, thus confirming the germline origin of the BLM variant and the somatic origin of the PALB2 variant. PALB2 c.3350+1G>T is located in the canonical splice site and predicted (spliceAI, maxEntScan) to result in the loss of the donor site, disrupting splicing and leading to the loss of RAD51 and BRCA2 binding domains of PALB2 and, thus, was classified as harmful.

**Figure 2:**
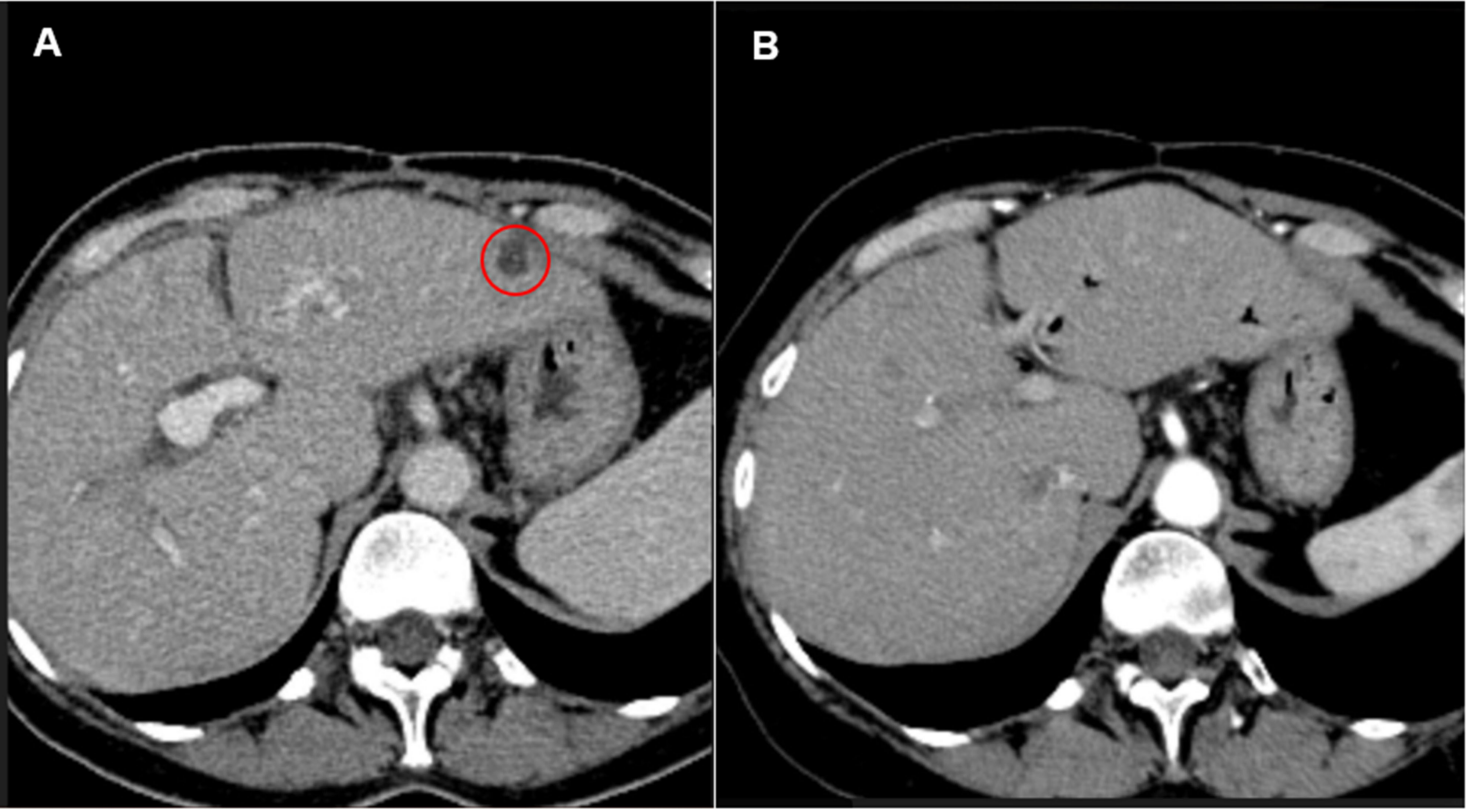
**(A)** CT scan of S2 liver lesion, 10 mm in greatest dimension before talazoparib treatment (March 2019), **(B)** CT scan of S2 of the liver after 25 months of treatment without visual evidence of the disease (April 2021).

**Figure 3:**
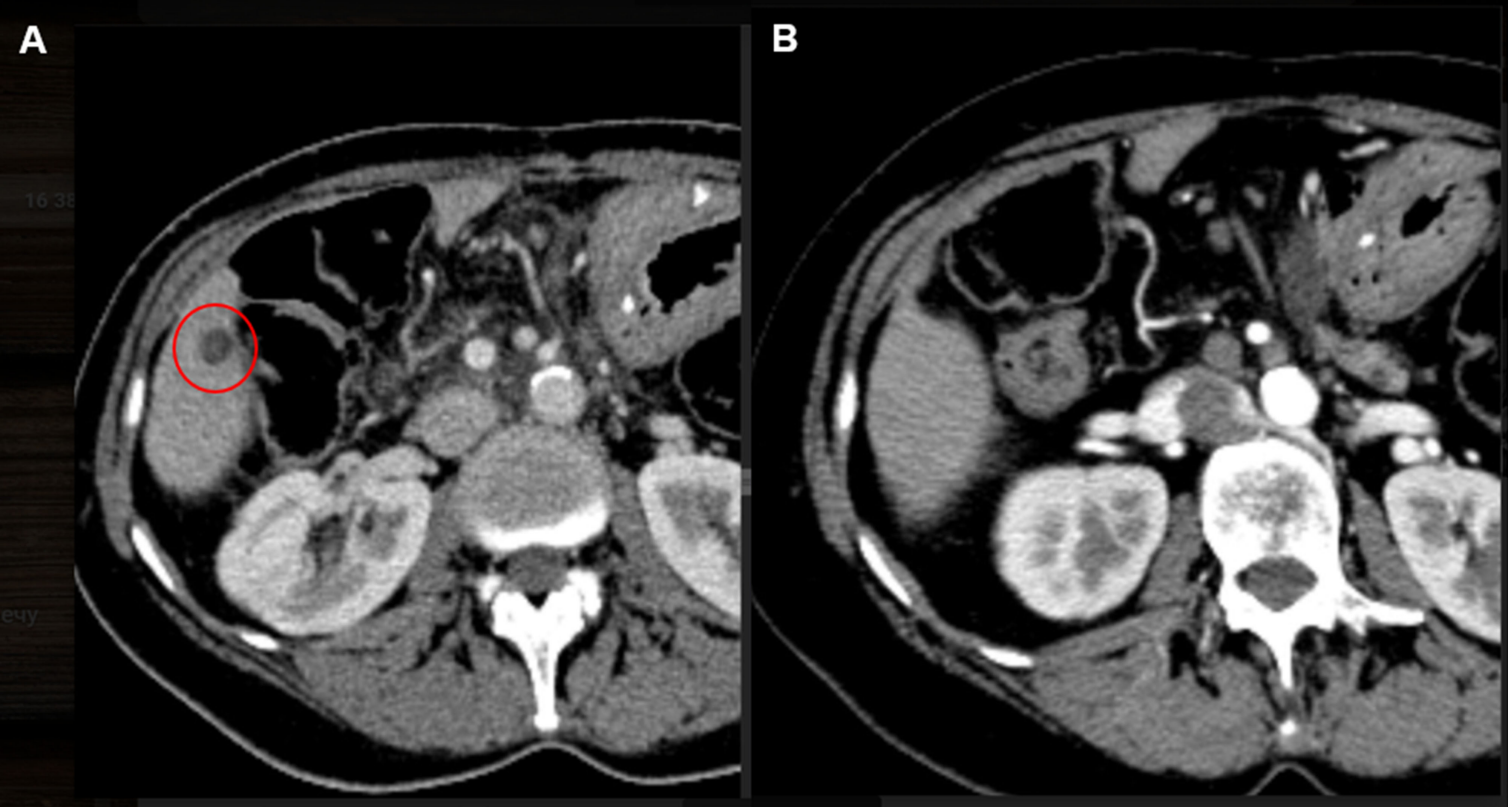
**(A)** CT scan of S6 liver lesion, 11 mm in greatest dimension before start of talazoparib treatment (March 2019), **(B)** CT scan of S6 of the liver after 25 months of treatment without visual evidence of the disease (April 2021).

With no standard therapeutic options left, the multidisciplinary tumor board has decided to start treatment with the PARP inhibitor talazoparib despite the previously registered disease progression on platinum-containing chemotherapy. The drug was provided by the expanded drug access program. The patient started to receive talazoparib at a dose of 1 mg once daily in March 2019. A complete clinical response had been achieved as of January 2021 (**Figures 2**, **3**): there were no signs of tumor lesions in the liver or signs of peritoneal carcinomatosis. The patient remains alive without clinical or radiological signs of disease progression after three years from the beginning of talazoparib treatment. The only toxicity affecting the quality of life of the patient is peripheral neuropathy, which has remained since first-line chemotherapy.

## Discussion

Pancreatic ductal adenocarcinoma is one of the most aggressive cancers. The median overall survival of patients is about 10–12 months, even with intensive regimens of chemotherapy (8–10). Attempts to increase survival rates have been made by improving the understanding of molecular pathogenesis, searching for targeted alterations, and implementating precision oncology. Several molecular epidemiology studies demonstrated that 10%–25% of PDAC patients have clinically actionable molecular alterations (11–13). The Know Your Tumor registry trial demonstrated that these clinically actionable molecular alterations in PDAC cancers may be successfully targeted with matched therapy (14, 15). Based on the trial results, across patients with clinically actionable alterations, median overall survival was significantly longer in patients who were administered with matched therapy compared with unmatched therapy (2.58 years vs 1.51 years, HR 0.42, 95% CI 0.26–0.68). Nevertheless, despite 26% of tumor profiles harboring actionable molecular alterations based on tissue analysis, across patients who were administered with molecular matched therapy, PARP inhibitors were given in 52% of patients matched to HR gene mutations (15). This defines the predominant role of DNA damage response genes and PARP inhibitors in the precision oncology of PDAC.

The POLO trial has made an important step forward in shifting the paradigm towards molecularly informed treatment decisions in PDAC patient management. Trial results showed a significant increase in median PFS in the olaparib treatment arm compared with placebo (7.4 months versus 3.8 months). More importantly, the trial demonstrated an impressive median duration of response in patients who had a radiological partial response to olaparib (24.9 months, compared to 3.7 months in the placebo arm), opening an unprecedented opportunity for durable clinical benefit for some PDAC patients (16). Although, considering the final overall survival results achieved in this study being similar between olaparib and placebo maintenance groups, the question of the clinical benefit of olaparib remains open, especially if compared with standard-of-care fluoropyrimidine maintenance strategies such as 5-fluorouracil/leucovorin and capecitabine regimens. Nevertheless, it is a very narrow patient population who harbors germline BRCA1/2 (17). The Phase II trial of rucaparib in germline or somatic BRCA1/2 or PALB2 mutations demonstrated treatment results consistent with the POLO trial, with a response rate in gBRCA2 and sBRCA2 subgroups of 41% and 50%, respectively. The difference of this trial was inclusion of patients with gPALB2 mutation. Although the number of such participants was modest (just 12 patients), the objective response to rucaparib therapy in this subgroup amounted to 50%. These results paved the way towards increasing the patient population who may benefit from PARP inhibitors by testing additional genes (PALB2) and additional alteration types (somatic in addition to germline). Recently, findings from two parallel nonrandomized trials of olaparib monotherapy in non-BRCA PDAC were presented (18). In addition to a lack of germline BRCA1/2 variants, eligible patients were required to have either an HR gene mutation or a family history of cancer. Across a total of 41 patients included, 24 had HR gene mutations (including ATM, PALB2, ARID1A, BRCA somatic, PTEN, RAD51, CCNE, and FANCB) and 15 had a family history of cancer. As a result, the response rate was only 2%, while disease stabilization was achieved in 72% of patients. Across patients with HR gene mutations, median PFS was 5.7 months compared to 1.9 months in a subgroup of patients enrolled based on family history alone (18). Lack of profit of family history of malignancies as an independent factor favoring clinical benefit from PARP inhibitors or platinum-chemotherapy is corroborated in phase II trial studying gemcitabine and oxaliplatin (19) and refuted in another retrospective analysis of overall survival of PDAC patients received diverse chemotherapy regimens in first-line setting (20). Such a discrepancy may come from the discordant methodology used to define the term “familial cancer,” pointing out that, firstly, gentle criteria used to define the family burden of cancer are not appropriate for PDAC patients and, secondly, thorough collection of family/personal history of cancer is of significant interest, so retrospective analyses would be possible.

Another major factor in selecting PDAC patients for PARP treatment is progression-free treatment duration on platinum-containing chemotherapy, which remains the first-line standard of treatment choice. In the POLO trial, eligible patients were required to have received at least 16 weeks of treatment with platinum-based chemotherapy without showcasing evidence of platinum resistance. The same inclusion criteria were used in the Phase II study of maintenance rucaparib in patients with germline or somatic variants in BRCA1/2 or PALB2, but the study protocol was amended to permit patients who had contraindications to platinum therapy to enroll without prior exposure. In the other two parallel nonrandomized clinical trials of olaparib for previously treated PDAC with HR gene mutations other than germline BRCA, platinum-resistance was defined as disease progression during or within 6 months of discontinuation of platinum-based therapy (18). In the latter trial, results demonstrated superior treatment results in the platinum-sensitive subgroup (median PFS of 2.2 months versus 4.1 months). HR gene mutations are associated with improved PFS when treated with first-line platinum-based chemotherapy compared to non-platinum-based (12.6 versus 4.4 months) (3). As a consequence, a durable response to first-line platinum chemotherapy may serve as a surrogate marker of a tumor with the so-called BRCAness phenotype. Such a surrogate marker may be useful considering the large number of genes involved in DNA repair, heterogeneity of effect of somatic, germline or even biallelic inactivation and the fact that BRCAness phenotype may be seen even when no mutations are found in HR genes (21). Nevertheless, about one fourth of patients who have a confirmed BRCAness phenotype still do not benefit from platinum chemotherapy (18). These results define a complex interconnection between the BRCAness phenotype, platinum-sensitivity, and the efficiency of PARP inhibitors. Further data are needed to shed light on optimal PARP inhibitor treatment selection, timing, and combinations for PDAC patients based on molecular profile, family or personal history of cancer, and response to platinum chemotherapy.

As for the selection between different PARP inhibitors, we have no data on direct comparison between any approved agent. Results of indirect analyses based on published phase III trials demonstrated no significant differences in the efficacy of talazoparib and olaparib for breast cancer patients (22), though talazoparib was more favorable for the BRCA mutated subgroup of patients (23). The latest result is consistent with *in vitro* studies demonstrating the higher PARP-trapping potency of talazoparib compared with other PARP inhibitors (6). Results of indirect analysis of the efficiency of olaparib, niraparib, and rucaparib for the treatment of platinum-sensitive recurrent ovarian cancer also demonstrated no statistically significant differences between these agents in terms of PFS (24). Nevertheless, toxicity profiles vary significantly for approved PARP inhibitors, which may play a major role in the selection of specific treatment for PDAC patients.

This case demonstrates an excellent response to talazoparib. Even though the patient had disease progression after six months of first-line platinum combination therapy (FOLFIRINOX), the clinical effect of talazoparib has persisted since treatment initiation in 2019. After 36 months of talazoparib therapy there is no evidence of disease and the drug is well tolerated. There is no toxicity from the drug that would lead to dose reduction or delays in treatment. However, peripheral neurotoxicity from first-line oxaliplatin-containing therapy remains a factor affecting the quality of life of the patient.

Patients who are exposed to long-term platinum-based chemotherapy develop neuropathy (25). Attention is usually given to neurotoxicity in PDAC patients, which is generally lower than in other cancer types, which is explained by the extreme aggressiveness of this cancer and lack of treatment options. However, once long-term clinical benefit occurs, long-term toxicity caused by first-line FOLFIRINOX may be brought to the forefront. This issue was confirmed in this study. Nevertheless, platinum-containing regimens such as FOLFIRINOX and gemcitabine + cisplatin continue to be among the main treatment options for metastatic PDAC, particularly in patients with HR deficiencies (26).

## Conclusion

This clinical case highlights the importance of NGS testing for PDAC patients. Targeted therapy based on the clinically actionable alterations detected by NGS may substantially improve survival in these patients. The selection of PDAC patients for molecular-matched therapy is still a matter of concern. Thus, firstly, molecular tumor boards would be preferred if any mutation is found; and secondly, broad molecular profiling would be beneficial. The latter implies not only testing for extended gene panels but also extending profiling beyond germline testing. An optimal strategy would be tumor NGS testing with the following validation of found alterations in blood tissue by Sanger sequencing if any potential germline variants are found.

## Data availability statement

The raw data supporting the conclusions of this article will be made available by the authors, without undue reservation.

## Ethics statement

The studies involving human participants were reviewed and approved by the Ethics Committees of P. Hertsen Moscow Oncology Clinical Research Institute. The patients/participants provided their written informed consent to participate in this study.

## Author contributions

AKa, AKo, and LB carried out the studies, participated in collecting data. MI, AKa, and OK drafted the manuscript. AF revised and commented on the draft. All authors contributed to the article and approved the submitted version.

## Conflict of interest

MI and OK were employed by Atlas Oncodiagnostics, LLC.

The remaining authors declare that the research was conducted in the absence of any commercial or financial relationships that could be construed as a potential conflict of interest

## Publisher’s note

All claims expressed in this article are solely those of the authors and do not necessarily represent those of their affiliated organizations, or those of the publisher, the editors and the reviewers. Any product that may be evaluated in this article, or claim that may be made by its manufacturer, is not guaranteed or endorsed by the publisher.
